# Validations and applications of markerless motion capture using OpenCap: a scoping review

**DOI:** 10.3389/fdgth.2026.1882536

**Published:** 2026-07-30

**Authors:** Xiaochen Zhang, Dunkai Mao, Haonan Shang, Liuchen Ma, Juncheng Jia, Jia Yu, Ming Zhang

**Affiliations:** 1 School of Physical Education and Sports, Soochow University, Suzhou, Jiangsu, China; 2 School of Computer Science and Technology, Soochow University, Suzhou, Jiangsu, China; 3 Department of Biomedical Engineering, Faculty of Engineering, The Hong Kong Polytechnic University, Hong Kong, China; 4 Research Institute for Sports Science and Technology, The Hong Kong Polytechnic University, Hong Kong, China

**Keywords:** biomechanics, kinematics, kinetics, rehabilitation, smartphone, sports

## Abstract

**Background:**

Recent advances in computer vision have substantially enhanced the accessibility and applicability of markerless motion capture systems. Among the available markerless motion capture platforms, OpenCap is a freely accessible, smartphone-based system that has received growing attention in biomechanics. Its applications have extended beyond controlled laboratory environments into field-based settings and beyond basic kinematic analysis to more complex clinical and sports-related applications. However, evidence regarding its concurrent validity, measurement accuracy, and reliability remains fragmented across study populations, movement tasks, and application contexts. This scoping review was designed to address two main research questions: (1) What evidence is available regarding the concurrent validity, accuracy, and reliability of OpenCap? (2) To what extent is OpenCap applicable across clinical, sports, and field-based settings?

**Methods:**

This review therefore synthesizes existing evidence on the validation and practical applicability of OpenCap. The scoping review was conducted in accordance with the PRISMA extension for scoping reviews (PRISMA-ScR). The systematic search identified 51 eligible studies, which included validation studies and applied studies using OpenCap.

**Results:**

Comparisons with reference-standard systems indicated that OpenCap performed most accurately for sagittal-plane measurements. Accuracy was generally higher for lower-extremity measurements than for upper-extremity measurements, in healthy individuals than in clinical populations, and during squatting and walking tasks than during jumping tasks.

**Conclusion:**

Future research should focus on expanding validation datasets across heterogeneous populations, improving tracking robustness under occlusion, and integrating multimodal sensing with large language model-assisted interpretation to support automated and context-aware biomechanical assessment.

## 1 Introduction

**Figure 1:**
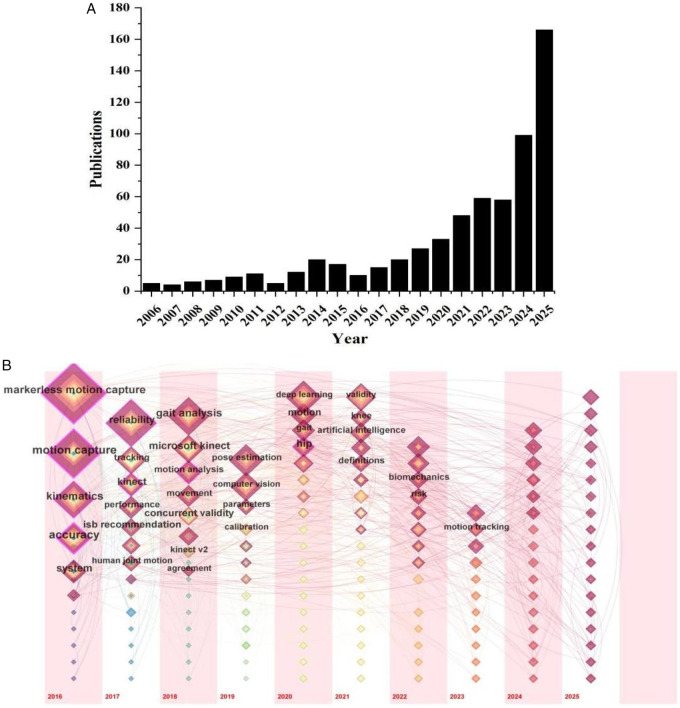
**(A)** Scifinder publication. **(B)** Citespace timeline.

Motion capture technology has long been regarded as an essential tool for understanding human movement (1), evaluating motor function (2), and informing training and rehabilitation protocols (3). Traditional marker-based motion capture systems provide high-accuracy three-dimensional (3D) kinematic information using reflective markers and multi-camera arrays. However, their dependence on costly hardware, controlled laboratory environments, and specialized operating procedures has limited their widespread adoption in clinical, home-based, and real-world sports settings (4). Early depth-sensor systems, such as Microsoft Kinect, enabled marker-free data acquisition but were constrained by restricted operating ranges, low frame rates, and limited robustness to occlusion. The emergence of deep learning-based two-dimensional pose estimation methods, such as OpenPose, marked an important shift from dedicated depth sensors toward conventional RGB cameras. At present, multi-view RGB video-based systems are widely used, and Theia3D and OpenCap represent prominent commercial and open-source platforms, respectively. In markerless motion capture, standard video is used as input, and computer vision and machine learning methods are applied to estimate body posture and joint motion directly from images (5), thereby enabling human movement to be quantified at lower cost and with shorter preparation time. These systems typically begin with 2D pose estimation, in which models such as OpenPose (6) and HRNet (7) are used to extract body keypoints that are subsequently reconstructed in three dimensions using multi-view triangulation (8). Marker augmentation models based on recurrent neural networks (9) have been used to further refine keypoint localization accuracy, thereby supporting downstream kinematic analyses. As a representative open-source platform, OpenCap integrates smartphone-based video acquisition with cloud-based processing. Its minimal configuration requires only two smartphones to collect data and generate 3D kinematic outputs, thereby lowering deployment barriers and improving portability compared with traditional systems (10). The present study conducted a systematic search of the Web of Science Core Collection and PubMed databases using “markerless motion capture” and “OpenCap” as keywords (2006–2025, limited to English-language publications). Following duplicate removal via EndNote, a total of 631 records remained after duplicate removal. The 631 retrieved records were only used for bibliometric analysis and excluded from the scoping review screening pool. Analysis of annual publication trends indicated that the field has moved from early development to rapid growth in recent years, reaching a peak of 165 publications in 2025 (Figure 1A). A keyword co-occurrence time-zone map was generated using CiteSpace 6.4.R1 for literature published between 2016 and 2025. The results indicate that the research trajectory has evolved through three successive stages: hardware validation, algorithm advancement, and application expansion. Core themes encompass the validation of technological accuracy, deep-learning-based pose estimation, and biomechanical applications. Research priorities have increasingly expanded to clinical rehabilitation and sports injury assessment, reflecting the growing integration of markerless motion capture into applied health and sports research (Figure 1B).

**Figure 2:**
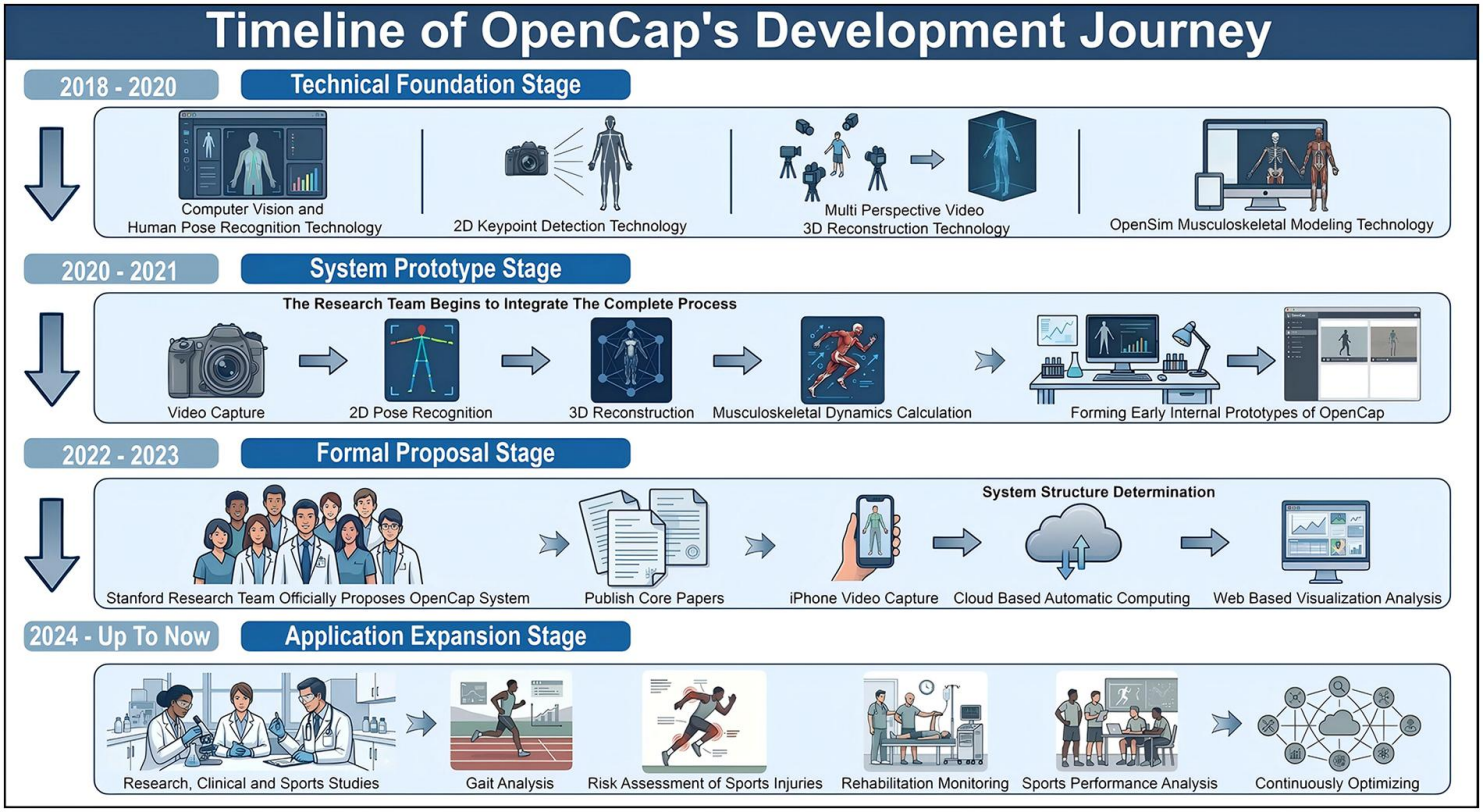
Timeline of OpenCap's development journey.

OpenCap (9) is an open-source, smartphone-based motion analysis system developed by the research team led by Professor Delp at the Neuromuscular Biomechanics Laboratory, Stanford University, and was publicly released in 2023, when its core system paper was published in PLOS Computational Biology. It estimates human kinematics and dynamics by integrating key components from computer vision and biomechanics, including 2D pose estimation models such as OpenPose and HRNet, multi-view 3D reconstruction, an LSTM-based marker augmentation model, and OpenSim-based musculoskeletal modelling (11). In this workflow, sparse 3D keypoints reconstructed from multi-view videos are transformed into anatomical marker trajectories compatible with OpenSim, thereby linking image-based pose estimation with biomechanical simulation. This end-to-end framework enables smartphone video acquisition, cloud-based computation, and web-based visualization of biomechanical outputs. Since its release, OpenCap has been increasingly applied to gait analysis, injury risk assessment, rehabilitation monitoring, and sports performance analysis (Figure 2).

**Figure 3:**
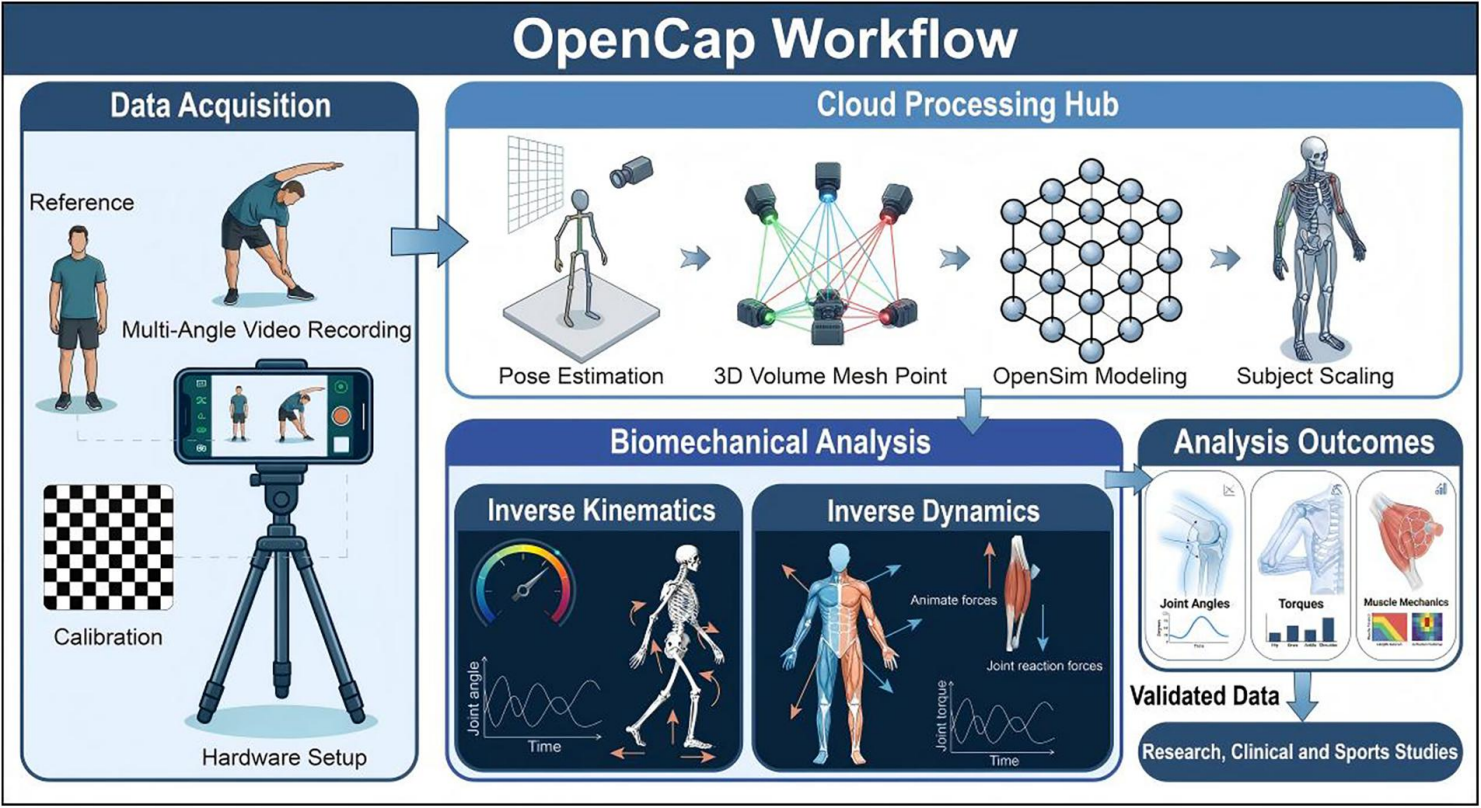
OpenCap working procedure.

OpenCap employs an edge-cloud software architecture (12) that links standardized mobile data acquisition with cloud-based biomechanical processing, as illustrated in Figure 3. During data collection, users perform camera calibration and record multi-angle videos through the mobile application or web interface, with standardized guidance on camera placement, filming distance, and movement tasks. After video upload, the cloud server detects and tracks body keypoints, reconstructs 3D keypoints using calibration parameters and multi-view geometric constraints, and applies LSTM-based marker augmentation to generate anatomical marker trajectories for OpenSim-based musculoskeletal modelling. The scaled musculoskeletal model is then used for inverse kinematics and inverse dynamics analyses, producing kinematic variables such as joint angles and dynamic variables such as joint moments, joint reaction forces, and muscle activation estimates. These outputs support clinical assessment, sports performance analysis, and biomechanical research.

OpenCap has been applied across a range of settings (13); however, evidence regarding the concurrent validity, measurement accuracy, and reliability of its outputs remains fragmented. Existing studies vary in population characteristics, task types, and validation metrics, which makes it difficult to determine the applicability of OpenCap across different settings. This review was designed to address two core research questions: 1) To what extent has the concurrent validity, accuracy, and reliability of OpenCap been validated in existing studies? 2) What is the practical applicability of OpenCap across specific domains? Concurrent validity refers to the level of agreement between OpenCap kinematic outputs and synchronous measurements from an established gold-standard motion capture system during matched movement trials; accuracy describes the proximity of OpenCap-derived kinematic values to ground-truth reference measurements, most frequently quantified using root mean square error (RMSE), while reliability denotes the consistency and repeatability of OpenCap kinematic measurements across repeated trials, testing sessions or raters, routinely evaluated via intraclass correlation coefficients (ICC). Through systematic searching and standardized screening, the available evidence on OpenCap validation and applicability was synthesized. The findings may inform future research and clinical implementation. This scoping review was conducted in accordance with the PRISMA-ScR reporting guidelines. In accordance with journal requirements for review articles, the research question was framed using the Population, Concept, Context (PCC) framework. Eligible studies were peer-reviewed, human-focused studies that used OpenCap and included quantitative validation studies and applied research. Comprehensive database searching, two-stage screening, and standardized data extraction were performed. Formal risk-of-bias assessment was not conducted, consistent with standard scoping review practice.

## 2 Methods

### 2.1 Search strategy

**Figure 4:**
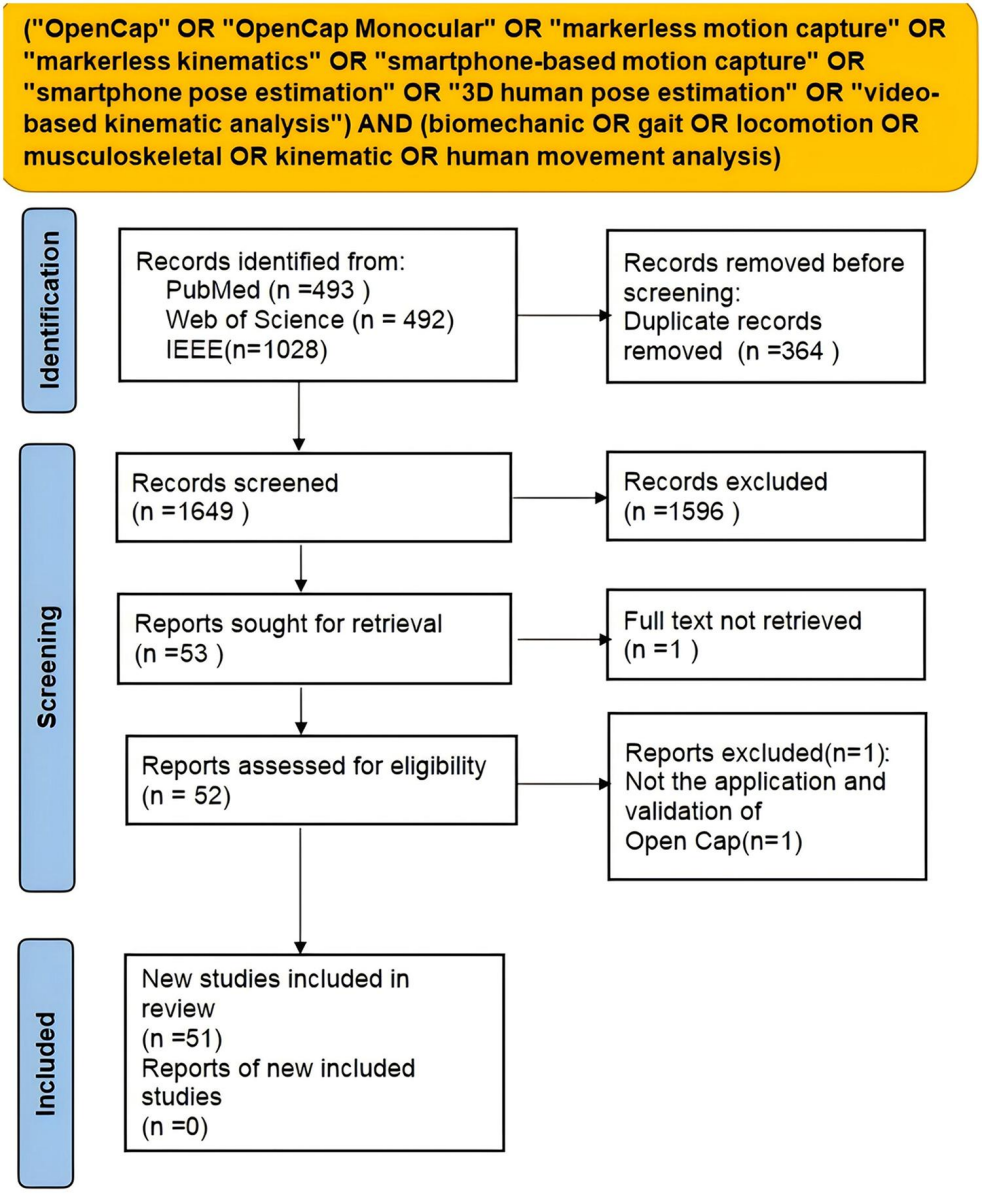
Review criteria: review flow chart.

We searched English literature across the Web of Science, PubMed and IEEE databases using subject headings. The search period covered all records from database inception to 27 March 2026. This scoping review, which is a type of systematic review, was performed in accordance with the PRISMA-ScR reporting guidelines. The complete search query was: (“OpenCap” OR “OpenCap Monocular” OR “markerless motion capture” OR “markerless kinematics” OR “smartphone-based motion capture” OR “smartphone pose estimation” OR “3D human pose estimation” OR “video-based kinematic analysis”) AND (biomechanic OR gait OR locomotion OR musculoskeletal OR kinematic OR human movement analysis) (Figure 4). OpenCap is the official name of the proprietary open-source markerless motion capture framework developed by the Stanford Neuromuscular Biomechanics Lab. To ensure retrieval comprehensiveness, backward citation tracking was performed using literature from the Stanford group (Uhlrich SD, Falisse A, Kidziński Ł, Delp SL) for complete article coverage.

### 2.2 Inclusion and exclusion criteria

Inclusion criteria: Studies must involve the application or validation of “OpenCap” to be included and retrieve from peer-reviewed journals. However, IEEE conference papers are also incorporated into the study in accordance with the research requirements. Exclusion criteria: (1) Studies focusing on animals; (2) Studies that did not utilize “OpenCap” were excluded; (3) Review articles were also excluded; (4) full text not retrieved; (5) Duplicate literature.

### 2.3 Data extraction

This scoping review was conducted in strict adherence to the Preferred Reporting Items for Systematic reviews and Meta-Analyses extension for Scoping Reviews (PRISMA-ScR) guidelines. The study protocol was finalized via full team discussion prior to study initiation, and no prospective public registration was undertaken. A standardized data extraction form was developed based on the predefined Population, Concept, Context (PCC) framework, aligned with the review's core research questions. Before formal extraction, a pilot test was performed on 3 representative included studies to refine field definitions and coding rules. The final extraction form was approved upon team consensus.

Five core data domains were extracted: (1) Study characteristics: first author, publication year, and document type; (2) Participant characteristics: population type (healthy/clinical populations), sample size, sex distribution, and testing setting; (3) OpenCap system configuration: number of cameras, sampling frame rate, and musculoskeletal model version; (4) Study protocol and outcomes: motion task types, reference gold-standard system, and core outcome measures (joint kinematic parameters, measurement error, agreement coefficients, etc.); (5) Study findings and conclusions: validity and reliability performance of OpenCap, key conclusions, reported limitations, and recommendations for future application.

Data extraction was completed independently in duplicate by two reviewers (Xiaochen Zhang and Dunkai Mao) with formal training in scoping review methodology, with respective expertise in computer science and sports biomechanics. Both reviewers contributed to full study design and the development of inclusion/exclusion criteria, and were fully familiar with the research objectives and extraction protocols. A two-stage disagreement resolution mechanism was implemented, with all discrepancies discussed prior to final documentation. First, the two reviewers re-examined relevant sections of the original publications and discussed discrepant items against pre-specified coding rules, prioritizing consensus through alignment with study criteria and source text verification. Unresolved discrepancies after discussion were escalated to a third senior reviewer (Jia Yu) with extensive experience in evidence-based research and domain expertise for adjudication. The third reviewer, who was not involved in routine extraction to avoid preconception, conducted blinded independent assessment based exclusively on predefined rules and original text; the adjudicated decision was deemed final. A total of 52 articles were selected for full-text review, of which 51 were ultimately included for data extraction. The basic characteristics of the included studies are summarized in Table 1.

**Table 1:** Summary: application fields, number of study, specific application, no. of participants, and references.

Application fields	No. of studies	Specific applications	No. of participants	Reference
Clinical Field	5	ACL	12 (6M6F)	(17)
			102	(41)
			10 (5 patients with ACLR and 5 healthy individuals)	(24)
			29 (22M7F)	(66)
			24 (17M7F)	(25)
	1	knee OA	83 (53 patients; 30 healthy individuals)	(18)
	9	neurological disorders	21	(19)
			129 (28FSHD; 58DM; 43 healthy subjects)	(40)
			22	(33)
			21 (9M12F)	(32)
			20 (10 healthy individuals and 10 patients)	(52)
			19 (12M7F)	(53)
			19 (9M10F)	(50)
			2 (1M1F)	(67)
			4 (3F1M)	(68)
	1	elderly individuals	26 (7 elderly participants and 19 young participants)	(61)
	1	technological innovation	12	(23)
	1	amputees	1 patient with amputation	(42)
Sports Field	1	spinal motion	10M	(27)
	3	both functional tasks and gait tasks	20 (18F2M)	(16)
			23 (10F13M)	(48)
			30F	(69)
	1	cycling task	10 (5M5F)	(14)
	11	gait movement	7M	(20)
			14 (8M6F)	(36)
			10 (6M4F)	(21)
			10 (6F4M)	(47)
			15 (7F8M)	(22)
			18 (12M6F)	(34)
			10 (5M5F)	(35)
			19 (7F12M)	(37)
			12 (4M8F)	(49)
			39 (24M15F)	(70)
			30 (19M11F)	(26)
	10	squats and jumps	19 (9F10M)	(28)
			12	(38)
			1M	(46)
			30	(15)
			33 (16M17F)	(39)
			15 (9M6F)	(30)
			22M	(31)
			32M	(71)
			6F	(72)
			40M	(73)
	1	walk, squat, sit-to-stand, drop jump	10 (6F4M)	(9)
	3	ball sports	10 (9M1F)	(29)
			19 (14M5F)	(57)
			14M	(74)
Field of Architecture	3	construction motions of construction workers	10M	(43)
		bimanual lifting and picking tasks	12 (3F9M)&13M	(44)
		symmetric and asymmetric manual lifting	21 (11M10F)	(45)

### 2.4 Data analysis

Narrative thematic synthesis was implemented to synthesize OpenCap's validation performance and application domains, in strict compliance with the PRISMA-ScR reporting guidelines. All extracted data items were exported to Excel for data cleaning, including standardization of categorical terms and identification of missing data. Two reviewers independently coded all text segments across the five domains covered by data extraction and synthesis. Preliminarily identified themes were further refined and harmonized through consensus discussions among the full research team. Both quantitative descriptive findings and qualitative thematic insights were integrated into a pre-established PCC framework to systematically identify gaps in the current research evidence regarding the measurement validity and reliability of OpenCap. To summarize the accuracy metrics across studies, RMSE values were extracted from each included study. For studies reporting RMSE as a range (minimum–maximum) rather than discrete values, the midpoint of the range was calculated and used as the study-specific mean RMSE for pooling purposes. Pooled summary statistics (median, Q1, and Q3) were then computed for each joint and anatomical plane. Median and interquartile ranges (IQRs) were calculated only for estimates derived from four or more studies; for estimates derived from three or fewer studies, only the range of reported RMSE values is presented, as the calculation of a meaningful median and IQR would be unreliable due to the limited number of data points. To avoid bias from extreme values, studies involving substantially different movement tasks [e.g., cycling (14), unplanned landings (15)] or technical issues [e.g., occlusion from safety bars (16)] were excluded from the pooled estimates for the corresponding joints. All data processing and statistical calculations were performed using SPSS 26.0.

## 3 Results

In the present review, 51 studies were included. Of these, 23 studies included validation analyses to assess the concurrent validity and accuracy of OpenCap. The remaining studies did not include dedicated validation assessments or direct comparisons with established reference-standard methods in the field and therefore could not be used to confirm the concurrent validity of the system.

These 23 eligible validation studies were identified through standardized screening of all extracted variables within our data charting framework: we screened for records explicitly reporting direct head-to-head comparisons between OpenCap and criterion reference systems for kinematic or kinetic outcome metrics. All 23 studies documented comparative validation results between OpenCap and gold-standard measurement tools for motion and force analysis, encompassing marker-based optical motion capture and force plates.

Across the included studies, OpenCap was implemented with 2, 3, or 4 iOS devices (iPhone or iPad) in accordance with each study's experimental design, with sampling frequencies set to 60 Hz (120 Hz and 240 Hz sampling modes are study- and device-specific, rather than inherent sampling frequencies). The reference gold-standard systems used for validation comprised marker-based motion capture systems (Vicon, SMART-DX, Nokov Mocap, OptiTrack), force platforms (Balance-B, Kistler 9287CA), and surface electromyography (sEMG). For the musculoskeletal models implemented in OpenCap, the majority were configured with a single degree of freedom (DOF) for the knee joint. The motor tasks assessed during validation included level walking, running, jumping, squatting, and cycling, among others. Detailed results regarding the concurrent validity and accuracy of OpenCap relative to the reference validation systems are presented in Table 2.

**Table 2:** Validation among various motor tasks (classified by application domain): setting, camera for markerless, sampling frequency, validation: Vicon/IMU, musculoskeletal model, motion tasks, kinematics/dynamics: concurrent validity, accuracy.

Setting	No. of camera & Fs (Hz)	Validation (Fs, Hz)	MSM	Motion tasks	Kinematics/dynamics	Concurrent validity	Accuracy	Study
LF	3 (60)	Vicon (100) & IMU (60)	OpenSim	standing, 3D spinal motions	thoracolumbar joint ROM	thoracolumbar angles: ICC (F/E = 0.03, LB = 0.57, AR = 0.28); segmental angle: *r* (F/E = 0.01, LB = 0.92, AR = 0.91)	RMSE = 0.27° (standing); RMSE = 98.79° (F/E)*;	(27)
LF	2 (60)	Vicon (250)	OpenCap DS	JLR, SLH, LVH	LJA	CMC = 0.76 (M)	MAE = 3.85°, RMSE = 4.44° (M)	(17)
LF	2 (120^#^)	Vicon (150)	OpenCap DS	Walking (WAC, WTC)	LJA	ICC: KOA (WTC0.63 > WAC0.5); HC (WTC0.75 > WAC0.64)	RMSE: KOA (WTC6.0° < WAC8.1°); HC (WTC4.7° < WAC6.7°)	(18)
LF	2 (60)	Vicon (120)	OpenSim	Walking (WAC)	pelvic and lower-limb kinematics	pathological < physiological	RMSE = 5.8 ± 1.8° (M); PE = 11.3 ± 3.9° (M)	(19)
rehabilitation assessment clinic	2 (60)	Balance-B with a force plate	OpenSim	unassisted standing	JA, CoM shake track, CoP	R = 0.39–0.94 (CoM vs. CoP)	RMSE = 3.20∼4.80 cm (CoM shake track); RMSE = 0.16∼0.22 cm/s (CoM shake speed)	(33)
LF	2 (60)	Vicon (200) & AMTI OR6 force plate (1,000)	AnyBody	Walking & running	LJA; segmental positions; GRF	angle: *p* ≥ 0.90 (SP); *p* = 0.74∼0.94 (M)	RMSE = 2.14∼7.08° (M angle); RMSE = 0.28∼1.32 BW (M GRF)	(29)
LF	3 (120^#^)	Mocap	OpenSim	SLS	knee and hip joints kinematics and dynamics	R^2^ = 0.77∼0.94	bias: 0.9°	(24)
laboratory treadmill	2 (60)	Vicon (240)	OpenCap DS	walking, running, DLS, CMJ, jump-landing	peak hip and knee angles	gait task < functional tasks	RMSE < 6°	(16)
laboratory cycle ergometer	4 (60)	Vicon (250)	OpenCap DS	cycling	LJA, Joint moment, Joint reaction force, Joint reaction moment	angle: *r* > 0.9 (Sag.) & *r* = 0.7∼0.9 (NSP)	knee VRF: RMSE = 37.7% BW (max); 90 rpm + high resistance: RMSE increased	(14)
LF	2 (60)	Vicon (100) & IMU (2,000)	OpenSim	walking	pelvic and lower-limb joint and ROM	pelvic: *r* = 0.1∼0.26; other: *r* ≥ 0.73	RMSE = 2.81∼8.20°	(20)
LF	2 (60)	Vicon (200)	OpenSim	SLS, sidestep cutting, side hopping, single-leg triple vertical hopping, DL-CMJ	trunk and lower-limb kinematics	marked bias variation, 50% of metrics compliant	RMSE = 6.3 ± 3.5°	(28)
LF	3 (60)	EMG (1,259.28)	OpenSim	squatting	lower limb muscle activation	normal > lean > obese	muscle activation: sEMG > MSK	(38)
LF	2 (60)	Vicon (150)	OpenSim	Walking (WAC, WTC)	spatio-temporal gait parameters, pelvic and lower extremity kinematic	gait parameters: *r* = 0.94∼1.00	RMSE = 4.5 ± 2.9° (kinematic); RMSE → 0 (gait parameters); WTC > WAC	(21)
LF	2 (60)	SMART-DX EVO (100)	OpenCap DS	jump-landing-jump with cognitive challenge	initial contact angles of lower limb joints and peak angles, similarity of time series	CMC ≥ 0.93.	SP > NSP	(15)
LF	2 (120^#^)	SMART DX 100 (100)	OpenCap DS	Walking (different speeds)	spatio-temporal gait parameters, JA (discrete, continuous), CoM	gait parameters: ICC ≥ 0.95, r ≥ 0.967; discrete joint angle: ICC = 0.482∼0.776, *r* = 0.54∼0.89	RMSE < 6 mm (CoM displacement); RMSE < 2° (all joint angles); Accuracy decreases with increasing speed	(22)
natural environment，LF	2 (60)	Mocap (100), force plate (2,000), EMG (2,000)	OpenCap	walking，squatting，sit-to-stand, DJ	joint rotation angles; joint moments; muscle activation	*r*^2^ = 0.65∼0.80	MAE = 4.5° (Joint angle); MAE = 12.3 mm (pelvic translation); MAE = 6.2% BW (GRF); MAE = 1.2% BW × ht (joint moment)	(9)
LF	3 (240^#^)	Vicon (100)	OpenCap DS	cricket bowling	key joint angles related to whole-body cricket bowling(discrete, continuous)	trunk and upper limbs < lower limbs	RMSE = 17.61 ± 7.72°	(29)
LF	4 (240^#^)	Kistler 9287CA (1,000)	OpenCap DS	CMJ, squat jump, BLJ, ULJ	GRF, direction of changes between arm swing/leg dominance conditions	*r* = 0.90–0.99 (CMJ & SJ); DJ landing peak force: *r* = 0.37∼0.67	Inaccurate variables: landing peak force and jump height	(30)
LF	2 (60)	OptiTrack (200)	OpenCap DS	overhead squat	angles of specific joints in different movement planes;	lower body: CMC = 0.93; upper body: CMC = 0.48; SP > FP > TP	lower body: RMSE = 5.45 ± 2.04°; upper body: RMSE = 36.7 ± 35.79°	(31)
LF	4 (240^#^)	Vicon (240) AMTI force plate (1,200)	OpenCap DS	DJ	frontal-plane knee angle; sagittal-plane knee joint moments; transverse-plane hip joint moments; GRF; Knee extensor moment asymmetry at IC	*r* > 0.9	RMSE = 6∼7°; moment: MAE = 5.6% (knee), MAE < 1% (hip); GRF: MAE = 6.5%	(25)
LF	4 (60)	BTS P6000 force plate (1,000)	OpenSim	squat at 60°/90°; step-up/down; lateral/posterior/lateral sliding/posterior sliding lunge	GRF	*r* = 0.82∼0.97	RMSE: 0.10 ± 0.03 BW; Timing error: 10.8 ± 8.0 ms	(69)
laboratory treadmill	3 (240^#^)	Vicon (200)	OpenCap + OpenSim	Running (different speeds)	CoM vertical displacement, lower limb kinematics, MTU length & lengthening velocity	overall consistency	RMSE = 3.6∼8.6° (angle); RMSE: <0.01 cm; MTU length differences: 0.01–0.03 L_0m_; MTU lengthening velocity differences: 0.11–0.26 L_0m/s_	(26)

Fs, sampling frequency; MSM, musculoskeletal model; OpenCap DC, OpenCap default settings; JLR, jump-landing-rebound; SLH, single-leg hop; LVH, lateral-vertical hop; SLS, single-leg squat; DLS, double-leg squat; CMJ, counter movement jump; DL-CMJ, double-leg counter movement jump; F/E, flexion/extension; DJ, drop jump; BLJ, bilateral landing jump; ULJ, unilateral landing jump; SJ, squat jump; LJA, lower-limb joint angles; JA, joint angles; L/B, lateral bend; AR, axial rotation; M, mean; KOA, knee osteoarthritis; HC, healthy control; WTC, walk towards camera; WAC, walk away from camera; PE, peak error; CoM, center of mass; CoP, center of pressure; SP, sagittal plane; NSP, non-sagittal plane; FP, frontal plane; TP, transverse plane; VRF, vertical reaction force; MSK, musculoskeletal modeling with OpenCap and OpenSim; IC, initial contact.

* Values marked with an asterisk denote flexion/extension outliers induced by occlusion.

# Denotes non-inherent frequencies of OpenCap.

Detailed joint-specific validation analyses of the included studies are presented in Table 3. Studies by (14–26) performed validation assessments exclusively for lower extremity joints, while studies including (9, 27–31) extended the validation scope to incorporate trunk and upper extremity joints in addition to lower limb evaluations. Extreme outlying values observed in the table stemmed from factors including motion occlusion and unplanned jump-land-jump tasks. As illustrated in Table 3, OpenCap demonstrates lower measurement accuracy for upper limbs compared to lower limbs. Its performance in the sagittal plane outperforms that in the frontal and transverse planes. Additionally, the system shows poor detection capability for the spine, and measurement errors rise sharply under occlusion or unplanned movements.

**Table 3:** Validation of concurrent validity and accuracy among various joints (lower limb: hip, knee, ankle; trunk; upper limb).

	Joint	Current validity	Accuracy RMSE^#^ (°)
Hip	SP	ICC = 0.665∼0.94; CMC = 0.85∼0.99; *r* = 0.64∼0.97; *r*^2^ = 0.81	5.99 (4.60, 6.90); *[7.12∼16.41 (16), 13.9∼31.3 (15)] (*n* = 13)
	FP	ICC = 0.21∼0.93; CMC = 0.61∼0.97; *r* = 0.02∼0.89; *r*^2^ = 0.79	3.93 (3.28, 4.87) (*n* = 12)
	TP	ICC = 0.25∼0.90; CMC = 0.51∼0.83; *r* = 0.37∼0.86; *r*^2^ = 0.76	5.22 (4.16, 5.62) (*n* = 11)
Knee	SP	ICC = 0.647∼0.98; CMC = 0.94∼0.99; *r* = 0.37∼0.97; *r*^2^ = 0.83∼0.94	6.07 (4.93, 7.36); *5.8∼14.5 (14) (*n* = 14)
Ankle	SP	ICC = 0.482∼0.80; CMC = 0.93∼0.99; *r* = 0.39∼0.91; *r*^2^ = 0.68	7.30 (5.99, 10.40) (*n* = 13)
	FP	*r* = 0.39∼0.88; *r*^2^ = 0.71	5.09 (3.78, 5.69) (*n* = 5)
	TP	*r* = 0.70∼0.90	×
Pelvic	SP	*r* = 0.20∼0.28; *r*^2^ = 0.78	5.10 (4.69, 5.85) (*n* = 4)
	FP	*r* = 0.10∼0.39; *r*^2^ = 0.75	4.48 (3.89, 5.14) (*n* = 4)
	TP	*r* = 0.31∼0.88; *r*^2^ = 0.74	3.55 (3.14, 4.16) (*n* = 4)
trunk	SP	ICC = 0.79∼0.92	2.8∼18.04 (*n* = 2)
	FP	ICC = 0.49∼0.88	6.84∼11.36 (*n* = 2)
	TP	ICC = 0.16∼0.79	14.47∼19.05 (*n* = 2)
Lumbar	SP	CMC ≥ 0.76	3.7∼6.9; *11.39∼101.15 (31) (*n* = 2)
	FP	CMC = 0.28∼0.42	3.9∼7.3; *4.39∼52.17 (31) (*n* = 2)
	TP	CMC = 0.24∼0.47	4.0∼7.6; *16.5∼19.34 (31) (*n* = 2)
Upper limb	SP	×	13.81∼30.02 (*n* = 2)
	FP	CMC = 0.28∼0.42	23.6∼52.17 (*n* = 2)
	TP	CMC = 0.24∼0.47	16.5∼36.4 (*n* = 2)

SP, sagittal plane; FP, frontal plane; TP, transverse plane; H, high applicability; M, moderate applicability; L, low applicability; *n*, number of included studies.

# Data are presented as median (Q1, Q3) for estimates derived from four or more studies, where Q1 and Q3 represent the 25th and 75th percentiles, respectively. For estimates derived from three or fewer studies, only the range of reported RMSE values is presented, as the calculation of a meaningful median and IQR would be unreliable due to the limited number of data points.

* Values marked with asterisk indicate abnormally large RMSE values reported in a small number of studies.

Among Table 4, 7 studies reported reliability outcomes, primarily encompassing inter-trial reliability, intra-session reliability, inter-system reliability, and conditional reliability. In this study, four dimensions of reliability were assessed to comprehensively characterize the measurement performance of the system. Inter-trial reliability denotes measurement consistency across repeated trials under identical conditions, reflecting both movement reproducibility and system stability. Intra-session reliability describes the overall consistency of multiple measurements within a single test session, indicating whether a single test outcome is sufficiently reliable for interpretation. Inter-system reliability quantifies the agreement between synchronous measurements from two distinct systems, serving as the core metric for verifying concurrent validity. Conditional reliability refers to measurement reliability across different test conditions, which defines the applicable scope and performance boundaries of the system across varied movement scenarios. The four reliability metrics exhibit a distinct performance pattern: excellent for lower extremity sagittal plane kinematics, poor for pelvic frontal and transverse plane measurements, and markedly diminished under complex movements and varying test conditions. While adequate for routine lower extremity assessments in research and clinical screening, the system presents clear limitations in multi-plane analysis, trunk and pelvic kinematics, and complex movement tasks.

**Table 4:** Reliability analysis: intra-trial, inter-trial, intra-session, and conditional reliability.

Reliability	Results	Study
Intra-trial reliability	pelvic-rot: *r* = 0.88; hip-flex: *r* = 0.97; knee-flex: *r* = 0.94; hip-add = 0.89; hip-rot = 0.73; ankle angle: *r* = 0.80; pelvic-list: *r* = 0.10; pelvic-tilt: *r* = 0.26;	(20)
Inter-trial reliability	70% of the extracted video-based movement features achieve moderate or higher reliability; *r* > 0.98	(40)
	inter-trial variability: markerless systems: 14.0%↑; physiological gait: 22.0%↑ (largest), equinus gait: 6.6%↑ (smallest); contributions of joints to inter-trial variability: pelvic-tilt, hip-flex	(32)
	RMSE = 2∼5° (M); segment lengths: ICC = 0.69∼0.97	(37)
	CoM vertical displacement: 0.4 cm (M); Joint angles: 1.1∼2.6° (M); MTU lengths: 0.3%∼0.6% L_0m_ (M); MTU velocities: 6%∼13% L_0m_/s (M)	(26)
	ICC = 0.70∼0.97	(39)
Intra-session reliability	ICC = 0.76∼0.94 (M); trunk-flex/ext: ICC = 0.79∼0.92, hip-flex/ext; ICC = 0.78∼0.92, hip-add/abd; ICC = 0.72∼0.88; knee-flex/ext: ICC = 0.25 (sidestep cut); ICC = 0.72 (peak angles); ICC = 0.64 (angles at IC)	(28)
	CoM vertical displacement: 0.3 cm (MV); Joint angles: 0.8∼2.2° (MV) MTU lengths: 0.2%∼0.4% L_0m_ (MV); MTU velocities: 5%∼11% L_0m_/s (MV)	(26)
	ICC = 0.79∼1.00	(39)
Conditional reliability	different clothing conditions: variability: walk↑, sit-to-stand→; discrete parameters: 1.2∼2.4°extra variability only in trunk flexion, pelvic tilt, subtalar joint motion; <1° for most; segment lengths: ICC = 0.45∼0.73	(37)

M, mean; ↑, increase; →, no change; MV, mean variability.

OpenCap has been applied across a wide range of clinical scenarios. In studies of knee sports injury and osteoarthritis, OpenCap has been used for biomechanical analyses of anterior cruciate ligament (ACL) injury and knee osteoarthritis (KOA). A previous study (17) demonstrated that OpenCap achieved high sagittal-plane measurement accuracy for ACL-injured knees and other lower-extremity joints (RMSE = 4.44°, CMC = 0.76, MAE = 3.85°), supporting the favorable reliability and precision of the system. Building on this work, another study (24) further validated and analyzed within-subject similarities and differences between the knee and hip joints during single-leg squats, revealing statistically significant yet closely correlated differences in knee and hip joint kinetics. In osteoarthritis research, the validity of the OpenCap system during walking tasks has been examined in both healthy participants and patients; with the exception of hip internal and external rotation, the RMSE and ICC of joint angles were significantly affected by cohort and walking direction. Smaller RMSE and higher ICC values were observed in healthy participants, and forward walking toward the camera yielded superior measurement performance compared with backward walking away from the camera.

In the field of neurological disorders, Horsak and colleagues validated OpenCap using pathological gait patterns mimicked by healthy adults in two consecutive studies (19, 32). The results for concurrent validity and accuracy were marginally higher than those originally reported by Uhlrich et al. (9), while the RMSE of knee sagittal plane angles was significantly elevated relative to conventional levels, which may be attributable to camera placement and the composition of the training dataset. In terms of reliability, OpenCap exhibited significantly increased inter-trial variability, whereas no significant difference was found in the overall inter-trial variability across different gait patterns. OpenCap also enables the estimation of the body's center of mass (CoM). Yan et al. (33) applied this function to children with cerebral palsy, and the results confirmed OpenCap-derived total CoM sway path showed strong correlation (R = 0.81) with the center-of-pressure (CoP) metric from the force plate, indicating good validity for quantifying overall quiet-standing sway in children with cerebral palsy. However, correlation coefficients across all balance parameters ranged from 0.39 to 0.94, with pronounced heterogeneity across individual metrics. This variability confirms that measurements from the two systems are not fully interchangeable and that systematic biases may persist. Accordingly, force plates—the gold standard—remain indispensable for high-precision, research-grade balance assessments and for investigating the mechanisms of pathology. Furthermore, the normalized symmetry index derived from OpenCap measurements offers a unique advantage over force plate-only assessments.

OpenCap has demonstrated broad applicability across diverse motor tasks and appears well suited for use in a wide range of movement analysis scenarios. Within human movement science, the most widely reported applications of OpenCap have centered on gait analysis, including assessments at varying walking speeds, including non-natural speeds (16, 22, 34, 35), during locomotion in different directions (21), and across diverse walking environments (36, 37). OpenCap has also been widely used for the biomechanical analysis of running (16, 34, 35). Validation work in this area has suggested that the system's sampling frequency may lead to the loss of fine-grained joint kinematic details under high-velocity conditions. Building on this identified limitation, a reliability analysis (22) was performed across a range of walking speeds; the results showed that the magnitude of kinematic underestimation by OpenCap increased slightly with increasing movement velocity, an effect primarily attributed to the system's sampling frequency being insufficient to fully capture high-frequency kinematic events during rapid movements. Consistent with these findings, analyses of cycling tasks using OpenCap have similarly shown that RMSE increased under high-velocity, high-resistance cycling conditions (14).

Beyond cyclic locomotor tasks, OpenCap has gained widespread adoption in the analysis of squatting and jumping tasks, which are core movements in sports performance and rehabilitation research. Within these paradigms, OpenCap has been leveraged to estimate muscle activation patterns (38) and ground reaction forces (GRF) (30), substantially extending its functional scope beyond pure kinematic analysis. Furthermore, independent studies by Lima et al. (28) and Turner et al. (39) have comprehensively validated the inter-session reliability, inter-system reliability, and concurrent validity of OpenCap during squat and jump tasks. Both studies consistently demonstrated that OpenCap exhibits good-to-excellent concurrent validity and reliability for the analysis of these movement tasks.

In addition, OpenCap has been validated for applications in spinal kinematic analysis, functional movement analysis, and biomechanical analysis of ball sports. Furthermore, machine learning models can be developed using the kinematic data acquired via OpenCap (40–42), which provides an efficient, objective, and quantitative approach for both clinical functional assessment and sports biomechanical analysis.

Furthermore, given its inherent low-cost advantage, excellent generalizability, and strong potential for large-scale implementation, OpenCap has recently been extended to industrial applications (43–45). In this field, it is primarily used for kinematic analysis of routine work-related movements of industrial workers, which further underscores its broad application prospects.

## 4 Discussion

Of the 51 studies included in this scoping review, only 23 performed direct quantitative validation of OpenCap against gold-standard motion capture systems (e.g., Vicon), while the remaining 28 lacked rigorous benchmarking. The lack of direct validation against gold-standard reference systems in over half of the included studies substantially weakens the overall evidence base supporting OpenCap's universal kinematic measurement accuracy. Furthermore, most trials enrolled limited participant numbers, introducing notable statistical generalizability constraints. Accordingly, robust interpretations of OpenCap's validity are only supported by the subset of gold-standard validated studies; broad extrapolations of its performance across various joint instability populations should be treated with considerable caution.

Compared with marker-based motion capture systems, OpenCap entails lower costs and requires far less time for device setup and calibration—approximately 1% of that for the gold-standard Vicon system (9)—thus rendering it suitable for large-scale implementation. In terms of measurement accuracy, the system demonstrates superior performance in the sagittal plane compared with the frontal and transverse planes, higher precision for the lower extremities than for the upper extremities, more reliable measurements during walking toward the camera vs. walking away from it, and better overall accuracy in healthy individuals relative to patient populations. The present review synthesizes studies in which OpenCap was used as a central tool to extend laboratory-grade biomechanical measurements (46) to clinical and sports training environments with reduced hardware barriers. In most studies, multi-view smartphone video recording was used and combined with pose estimation and 3D reconstruction algorithms to output 3D joint kinematics. OpenCap can also be integrated with musculoskeletal modeling packages such as OpenSim (47) to estimate kinetic variables, including joint moments, GRF, and muscle activation (14, 38, 47), thereby providing a more comprehensive analytical foundation for injury risk screening and rehabilitation monitoring. Additionally, OpenCap may reduce issues related to marker loss and occlusion, with error patterns that have been characterized. Assessments of the lower extremity and sagittal-plane movements are generally more robust, and overall, the system provides clinically interpretable 3D biomechanical metrics within acceptable error bounds.

Compared with other markerless motion capture technologies, including Theia3D, stretch-sensor-based systems, and monocular vision analysis approaches, OpenCap is associated with distinct technical trade-offs and application advantages. Existing studies (48, 49) have shown that Theia3D achieves higher measurement accuracy than OpenCap; however, it relies on a multi-camera array for kinematic measurement and estimation, resulting in higher hardware costs and greater deployment complexity. Although stretch-sensor-based systems have a simple configuration and low cost (46), they have inherent limitations in the measurable range of joint range of motion (ROM), provide lower measurement accuracy than OpenCap, and are therefore unsuitable for high-precision research. Compared with monocular vision-based motion analysis methods (47, 48, 50), OpenCap provides broader spatial coverage. Furthermore, its integrated multi-camera triangulation algorithm and LSTM model help address the core limitation of insufficient spatial information inherent in single-view systems, thereby improving the estimation accuracy of joint kinematic angles. In terms of application breadth, OpenCap, as an open-source system, has shown promising application potential across a wide range of research and practical fields.

### 4.1 Clinical applications

In clinical settings, OpenCap has been applied to the acquisition and analysis of spatiotemporal gait parameters, as well as kinematic and kinetic data, in populations including patients undergoing ACL rehabilitation, those with KOA, and individuals with neurological disorders. Additionally, an error within 5° is widely accepted as the acceptable threshold for joint angle measurements in clinical settings (19, 51).

In studies of patients undergoing ACL rehabilitation (17, 24, 41), the musculoskeletal models used in two studies (17, 24) were limited to a single degree of freedom (DOF) for the knee joint. The native OpenCap software does not include a module for calculating frontal-plane knee motion (e.g., varus/valgus angulation). However, the stability of frontal-plane knee motion is a critical metric for evaluating rehabilitation outcomes and guiding treatment regimen adjustments, and it is directly related to short-term recovery quality and long-term prognosis after ACL reconstruction. This limitation may result in an incomplete assessment of recovery status in patients with ACL injury. Even when frontal-plane knee angles were derived in one study (25) by integrating the default OpenCap-output musculoskeletal model with the underlying OpenSim framework, the resulting errors exceeded the clinically acceptable threshold, indicating a critical need to improve the accuracy of frontal-plane motion estimation. Similarly, in a study that analyzed motor function in patients with KOA (18), the current OpenCap-generated musculoskeletal model was unable to account for joint deformities commonly present in clinical populations. Coupled with the incomplete coverage of the existing OpenCap training dataset, this may lead to notable limitations in the identification of abnormal knee motion.

In studies related to neurological disorders, experiments were conducted in which healthy individuals mimicked three types of pathological gait patterns (19, 32, 50). The OpenCap training dataset is predominantly composed of activities and movements from able-bodied individuals, whereas pathological gait exhibits exaggerated femoral and tibial rotations compared with physiological gait. This discrepancy may limit the ability of OpenCap to accurately estimate the joint kinematics of pathological gait. Furthermore, pose estimation for different joints may introduce additional errors beyond the natural variability of the signal, leading to increased inter-trial variability in OpenCap measurements and the introduction of spurious individual outliers during participant identification. However, because the abnormal gait patterns in the aforementioned studies were mimicked by healthy individuals, they may not fully reflect the gait variability inherent in real patient populations. Future research should expand the range of participant populations and apply OpenCap to broader cohorts of patients with genuine pathological gait abnormalities.

A separate body of work has examined clinical patients with neurological disorders (33, 40, 52, 53). Among these studies, OpenCap was applied to assess balance ability in children with cerebral palsy (CP), primarily by comparing the CoP with the CoM measured using force plates, thereby expanding the application of OpenCap into a new biomechanical domain (33). However, the standard OpenCap musculoskeletal model, namely the OpenSim LaiUhlrich2022 musculoskeletal model, was developed on the basis of the anthropometrics of typically developing individuals. Children with CP often present with altered segment lengths and muscle volumes, which may reduce the accuracy of kinematic and kinetic estimates. Similarly, in another study (52), distinct gait strategies were observed between healthy individuals and patients with neurological disorders; thus, the standard musculoskeletal model may not accurately evaluate or predict patients' movements, resulting in reduced estimation precision. Future research should focus on developing algorithms to improve skeletal tracking accuracy, establishing disease-specific musculoskeletal models tailored to adult patients with stroke, Parkinson's disease, and other neurological conditions, and developing pediatric-specific models for children with CP that incorporate differences in height, body weight, and muscle proportions through modified segment length and muscle volume parameters. These optimizations may improve the accuracy of kinematic and kinetic estimates in pediatric populations, thereby providing more reliable information for individuals across different age groups and clinical conditions.

In clinical settings, patient safety should be considered the primary priority. Before gait analysis is initiated using the OpenCap system, individuals are required to maintain a brief static posture, such as neutral standing or another fixed posture, typically for 5 s, for model scaling. This process requires the individual to be captured from two camera views while maintaining the static position without assistance, which may pose a substantial challenge for patients with impaired balance function or pediatric populations. Therefore, when OpenCap is applied in clinical practice, video capture protocols should be adjusted according to each patient's physical condition to ensure safety. However, the current OpenCap system lacks support for multi-person recognition, and occlusions caused by assistive devices, such as parallel bars or crutches, may compromise its measurement accuracy. Accordingly, future development of protocols that support multi-camera setups capable of tracking body keypoints from posterior views, or software updates that enable multi-person recognition, could substantially expand the usability of the OpenCap system for a broader range of patients, including those with neurological disorders who rely on assistive devices.

### 4.2 Sports applications

In the field of sports biomechanics, the markerless motion capture system OpenCap has been increasingly adopted for the acquisition and analysis of 3D kinematic and kinetic data across a diverse range of functional and sporting tasks, including spinal motion, standardized functional assessments, overground and treadmill gait, cycling, squat and jump movements, and dynamic skills in individual ball sports. No established error threshold has been defined for kinematic measurements during dynamic movements.

With respect to spinal biomechanics, a study by Wang et al. (27) compared spinal kinematic measurements in healthy adults obtained using three modalities—OpenCap-only, an optimized OpenCap–inertial measurement unit (IMU) integrated system, and IMU-only—with gold-standard multi-camera optical motion capture used as the reference. The authors reported that the lowest RMSE across all conditions was observed for OpenCap-only measurements under static conditions, indicating greater static accuracy relative to the other modalities. However, the largest kinematic estimation errors were also recorded for OpenCap during dynamic tasks involving substantial body-segment occlusion. This dichotomy suggests that although OpenCap performs well in static pose estimation, it may be susceptible to substantial stochastic errors during dynamic movements when body segments are occluded or video input is unstable, underscoring the need for further optimization of the system's data acquisition and pose-tracking pipeline.

For gait analysis, a notable inconsistency was observed between the findings of Svetek et al. (16) and other relevant gait validation studies (20–22, 34–36, 47–49): specifically, Svetek et al. (16) reported significantly higher RMSE for sagittal plane hip kinematics during treadmill gait. This discrepancy was primarily driven by occlusion from the treadmill safety bars, which were positioned at the participants' mid-abdominal level and obscured the hip joint from the two-camera OpenCap setup. In contrast, the gold-standard Vicon optical motion capture system utilized multiple cameras with unobscured multi-angle views of the hip joint, eliminating the impact of the safety bars. As the safety barriers could not be removed from the treadmill environment, this occlusion effect was unavoidable with the two-camera configuration, directly driving the increased error and variance in sagittal plane hip measurements. These findings indicate that adjusting the number, placement, and angles of cameras to mitigate occlusion from environmental structures can substantially improve the accuracy of OpenCap's kinematic measurements across all anatomical planes. Horsak et al. (37) reported relatively high absolute minimum detectable change (MDC) values for trunk, pelvic, and hip kinematics in the sagittal plane, a finding primarily explained by the substantially larger native ROM of the trunk-pelvis-hip complex in this plane relative to the frontal and transverse planes. Importantly, while larger absolute angular errors contribute to higher absolute MDC values, the relative error (normalized to ROM) is inherently lower for movements with larger excursion. To account for ROM-dependent differences in MDC, normalized MDC (MDC% = MDC/ROM × 100%) is recommended for reliability assessments. With this normalized index, sagittal plane measurements demonstrate low MDC% despite higher absolute MDC values, indicating excellent reliability in clinical and research settings. During overground gait at self-selected natural speed, Martis et al. (21) compared OpenCap's performance between two walking directions: walking toward the cameras (WTC) and walking away from the cameras (WAC). The authors reported significantly higher kinematic errors during WAC gait, driven by three core limitations: first, OpenCap's underlying OpenPose-based pose estimation algorithm has inherently lower recognition accuracy for anatomical keypoints on the posterior aspect of the body; second, the musculoskeletal model pipeline is not optimized for movements where anterior body landmarks are obscured from the camera view, leading to error propagation and amplification in joint angle calculations; and third, insufficient video sampling frequency and spatial resolution further degrade keypoint tracking performance during WAC gait, reducing the quality of input data for kinematic modeling.

In a validation study across a spectrum of walking velocities, de Borba et al. (22) demonstrated that OpenCap's kinematic measurement errors escalate incrementally with increasing gait velocity—a trend congruent with observations reported for high-velocity dynamic movements, including explosive jumping tasks. OpenCap's relatively low sampling rate (max 240 Hz in existing work) fails to capture high-frequency kinematic transients during fast movements. This is particularly relevant for kinematic parameter estimation: while (30) noted that the minimum sampling frequency recommended for resolving high-frequency components of GRF profiles (including take-off and landing events, and peak impact forces during landing) is >1,000 Hz, consistent with established guidelines in sports biomechanics (54–56), it is imperative to clearly delineate the applicable boundaries of sampling frequency requirements between markerless motion capture systems and direct force measurement techniques. The aforementioned >1,000 Hz sampling requirement applies exclusively to direct GRF acquisition using force plates. Force signals exhibit an extremely short rise time during the ground contact impact phase, such that high sampling rates are essential to fully capture peak loads and the morphology of force-time curves. For OpenCap, by contrast, its maximum sampling rate of 240 Hz applies to kinematic data acquisition; its GRF outputs are indirectly derived from kinematic data via inverse dynamics coupled with musculoskeletal models, rather than being measured directly. As such, the sampling frequency threshold established for force plates cannot be directly extrapolated to this markerless motion capture system. Nevertheless, the 240 Hz kinematic sampling rate is not without limitations. For high-speed transient movements (e.g., vertical jump landing, sprint ground contact), the loss of high-frequency kinematic details may compromise the accuracy of captured joint trajectories, which in turn indirectly degrades the estimation accuracy of GRF during transient phases including ground contact and toe-off. Accordingly, these high-frequency GRF characteristics cannot be reliably estimated from kinematic data sampled at lower frequencies. Despite these speed-dependent limitations, de Borba et al. (22) reported an RMSE of <2° for all evaluated joint ROMs, a finding attributable to the fact that all measurements were restricted to the sagittal plane. This result further corroborates the existing consensus that OpenCap achieves its highest measurement accuracy for sagittal plane movements, and underscores the critical need for future advancements to improve its performance in capturing non-sagittal plane kinematics and high-velocity dynamic movements.

A study by Farber et al. (14) validated OpenCap for cycling biomechanical analysis using a four-camera setup. This configuration was adopted to account for the inherent complexity of cycling movements and to comply with recommended camera-deployment specifications for such tasks. However, the authors reported that OpenCap was unable to accurately capture subtle kinematic deviations in the frontal and transverse planes, in which motion amplitudes are inherently small during cycling. To address this limitation, future research should prioritize increasing the density of tracked anatomical keypoints, particularly for the lower extremity and pelvis, to improve the detection of small but biomechanically meaningful movements in non-sagittal planes. Furthermore, cycling-related research should be extended beyond controlled laboratory settings—where investigations typically rely on manipulated speed and resistance parameters—to naturalistic outdoor riding environments. This field-based validation may facilitate iterative refinements to OpenCap's robustness in unconstrained conditions, thereby supporting more reliable and widespread deployment of the system in field-based sports biomechanics and occupational ergonomics research. Two independent studies have evaluated the inter-session reliability of OpenCap for dynamic functional tasks (28, 39). A key discrepancy between the findings was that one study (28) reported poor inter-session reliability for knee kinematics during the single-leg triple vertical hop task. Importantly, the authors attributed this poor reliability to substantial between-participant heterogeneity in movement execution and task performance, which resulted in high data dispersion, rather than to inherent measurement limitations of the OpenCap system itself. One study (38) validated OpenCap-derived muscle activation estimates against reference-standard sEMG measurements. Importantly, muscle activation is not directly computed by OpenCap; instead, captured kinematic data are used to drive static optimization and inverse dynamics analyses within the OpenSim framework to generate muscle activation estimates. This indirect estimation pathway may introduce systematic discrepancies between directly measured sEMG and model-derived muscle activation. To mitigate this issue, future studies should focus on optimizing the underlying musculoskeletal model and integrating sEMG data as a biological constraint to refine OpenCap's muscle activation and kinetic estimates. Another study (30) demonstrated that OpenCap achieved the highest accuracy for GRF and kinematic estimates during the propulsion phase of countermovement jumps and squat jumps. Because the propulsion phase is the primary period of interest for sports performance profiling and neuromuscular fatigue assessment, this finding supports the utility of OpenCap-derived GRF estimates for these field-based applications. However, as noted previously, the system's limited maximum sampling frequency may prevent accurate resolution of high-frequency transient events during jump landing and takeoff. To address this limitation, future advancements in OpenCap's data acquisition hardware and software pipeline are needed to support higher sampling frequencies. Furthermore, given OpenCap's ability to estimate whole-body kinematics and GRF, these outputs can be used to derive a wide range of secondary biomechanical variables, such as joint power, work, and stiffness, for specific research applications using established biomechanical principles and physics-based equations. One study (31) evaluated the concurrent validity and between-system agreement of OpenCap for upper- and lower-extremity joint kinematics and reported poor agreement for the upper extremity, characterized by low coefficients of multiple correlation (CMC) and large, highly variable RMSE values. This poor upper-extremity performance may be explained by three core limitations: first, inherent constraints in OpenCap's pose estimation algorithm and recurrent neural network (RNN) model, which are optimized for lower-extremity and trunk tracking rather than complex upper-extremity movements; second, insufficient representation of large-amplitude, multiplanar upper-extremity movements in the system's training dataset, which means that shoulder motions exceeding the ROM represented in the training data cannot be accurately estimated; and third, the absence of scapulothoracic kinematics in the default musculoskeletal model, in which the glenohumeral joint is simplified as a 3-degree-of-freedom ball-and-socket joint, thereby limiting its ability to capture the physiological complexity of shoulder girdle motion. Consistent with these limitations, another study (29) reported the lowest measurement accuracy for shoulder kinematics, specifically shoulder elevation plane and axial rotation, during cricket bowling, a dynamic task characterized by extreme, high-velocity upper-extremity movements. Collectively, these findings highlight the need for future research to expand and refine the upper-extremity training dataset, increase the density of tracked upper-extremity anatomical keypoints, and develop a more physiologically accurate shoulder girdle model to improve upper-extremity tracking performance.

Within individual ball sports biomechanics, two studies have applied OpenCap to dynamic sporting skills: one study (29) analyzed upper-extremity kinematics during cricket bowling, whereas another (57) evaluated lower-extremity landing kinematics during standing vertical jumps in a competition volleyball-court setting rather than in a controlled laboratory environment, providing valuable field-based validation. However, an important limitation for team-sport applications is that the current OpenCap pipeline supports only single-person motion capture and cannot simultaneously track multiple players, such as setters, spikers, and defenders, within the same capture volume. This constraint substantially limits the utility of OpenCap for comprehensive team-sport analysis, underscoring the need for future software updates to enable multi-person tracking and full-team biomechanical assessments. Notably, both ball-sport studies reported substantial rates of invalid data and excluded trials when OpenCap was used. One study (29) reported an overall successful trial rate of only 56% (267 of 473 total trials), with failures attributed to the fact that OpenCap's pose estimation algorithm had not been trained on the specific movement patterns of cricket bowling, which are characterized by extreme high-speed upper-extremity rotation and large-amplitude trunk flexion. This lack of task-specific training may lead to frequent keypoint-recognition errors during periods of joint occlusion, such as shoulder and elbow occlusion by the trunk during the front foot contact (FFC) phase of bowling. Similarly, another study (57) excluded approximately 30% of all trials from analysis because of OpenCap data-processing failures, which were primarily associated with lower-extremity segment overlap during jump landing, resulting in occlusion in one of the two camera views and failed tracking. These high trial-exclusion rates were not isolated findings, as similar data-loss issues have been reported across the broader OpenCap validation literature. In one study (58), only five valid datasets could be retrieved from a larger sample because of repeated data-upload failures through OpenCap's web-based server, whereas another study (15) reported that approximately 10% of all recorded trials were discarded because of synchronization failures or internal processing errors within the OpenCap pipeline. Collectively, these technical issues may result in the loss of valuable collected data, increased research costs, avoidable participant burden, and delays in study timelines, representing important bottlenecks that should be addressed to expand the real-world utility of OpenCap. To mitigate occlusion-related trial failures in field-based sports settings, optimized camera-placement strategies should also be explored to accommodate environmental structures, such as court nets, and participant movement trajectories within the capture volume.

### 4.3 Limitations

An important limitation of this scoping review is that the literature search was restricted to English-language publications; therefore, OpenCap-related studies published in Chinese and other languages were not included, which may have led to an underestimation of the practical application and validation progress of this system in Asian and non-English-speaking countries. Consistent with methodological guidelines for scoping reviews, review articles were excluded, and only original research was included; this precluded the systematic synthesis of comprehensive conclusions from existing secondary research and therefore limited the coverage of the current evidence base. The included studies varied considerably in experimental setup, study population, motor tasks, number of cameras, and sampling frequency. Moreover, different statistical metrics were used across studies to report concurrent validity, including the intraclass correlation coefficient, coefficient of multiple correlation, RMSE, and Bland-Altman limits of agreement. Such heterogeneity in measurement metrics restricted quantitative cross-study comparisons, making it difficult to establish a unified quantitative evaluation of the overall accuracy of OpenCap across different tasks and populations. Healthy participants constituted the majority of the included samples, whereas studies involving pediatric, geriatric, and other special clinical populations were limited, making it challenging to extrapolate the current findings to these groups. The OpenCap system is undergoing continuous iterative development, and the software versions used in some early validation studies have since been updated. Technical differences between different versions were not systematically distinguished in this review, which may affect the applicability of some accuracy-related conclusions. Finally, this review mainly included peer-reviewed journal articles and conference papers, whereas preprints and unpublished data were not systematically included, which may have introduced a risk of publication bias. Consequently, the available evidence may overestimate the accuracy and applicability of OpenCap.

## 5 Future perspectives

### 5.1 Practical constraints in real-world implementation

The translation of OpenCap from structured laboratory environments to real-world clinical and field-based settings remains constrained by several practical factors. Although standardized acquisition protocols can be reproduced under controlled conditions, spatial limitations (40), unstable lighting conditions (43), and the involvement of non-specialist operators may compromise calibration quality. The absence of real-time feedback during data acquisition also makes it difficult for operators to determine whether the collected data are suitable for subsequent analysis. In addition, subtle variations in camera placement across repeated assessments may introduce systematic bias (37), while the lack of standardized cross-setting error normalization may limit the comparability of longitudinal data and pooled multi-centre analyses (59).

Several population-specific factors further complicate implementation. Patients who rely on assistive devices may have difficulty maintaining the static calibration posture required by the system, and these devices may occlude key joints, thereby affecting the accuracy of 3D reconstruction (29, 60). OpenCap currently provides limited processing solutions for such conditions. Evidence in paediatric and older adult populations also remains scarce (61). Atypical body proportions in children and highly individualized gait patterns in older adults may place additional demands on the generalizability of the underlying models (33).

Sport settings present another set of practical challenges. Variable lighting conditions, limited capture space, venue-specific constraints, and mutual occlusion among athletes can make standardized camera configurations difficult to maintain. The current single-person capture workflow also restricts the application of OpenCap in multi-person team sport scenarios. Moreover, sport-specific technical analyses involving upper-limb-dominant movements, such as throwing and striking, remain limited by insufficient estimation accuracy at the upper-extremity joints. Collectively, these factors define the current implementation boundaries of OpenCap and should be considered when interpreting its outputs across clinical, sport, and field-based contexts.

### 5.2 Future research recommendations

Future work should extend beyond technical validation and further examine how OpenCap can be implemented safely, reliably, and consistently across real-world clinical, sports, and community-based settings. The development of population- and task-specific datasets may improve the system's generalizability to pediatric and older adult populations (61), individuals after anterior cruciate ligament reconstruction (14), patients with knee osteoarthritis, children with cerebral palsy, and people with neurological disorders (33). In parallel, refinements to musculoskeletal models and joint-level outputs, including the extension of selected joints from single-degree-of-freedom to multi-degree-of-freedom representations, may enhance the interpretability of OpenCap-derived outcomes for rehabilitation assessment, injury risk evaluation, and longitudinal monitoring (62).

Implementation-oriented research is also needed to strengthen the robustness and usability of OpenCap under less controlled conditions. Optimized camera placement, improved keypoint detection, and multimodal integration with inertial measurement units (IMUs) (63), plantar pressure sensors, or other wearable technologies may help mitigate errors arising from occlusion, unstable lighting, environmental constraints, and complex movement patterns (64). In addition, automated reporting tools, including large language model-assisted interpretation, may facilitate the translation of structured biomechanical outputs into more accessible clinical and performance reports (65). Together, these developments may help clarify the conditions under which OpenCap can be deployed with confidence and support its integration into routine digital health workflows and field-based biomechanical assessment.

## 6 Conclusion

In conclusion, this scoping review systematically mapped and synthesized evidence on the concurrent validity, measurement accuracy, reliability, and applicability of OpenCap across different settings. The progress in benchmarking OpenCap against reference-standard kinematic and kinetic measurements was summarized, and its current applications and practical value across clinical, sports, and industrial settings were clarified. Existing validation studies have consistently highlighted a core strength of OpenCap: its low-cost, field-deployable workflow, which enables feasible human biomechanical analysis in out-of-laboratory environments. However, current evidence primarily supports the use of OpenCap for lower-extremity and sagittal-plane analyses in healthy populations, while evidence remains limited in several clinically relevant contexts. This review also identified key technical and practical limitations of the system in its current form, including its limited capacity to output multi-DOF kinematic parameters and the reduced stability of data acquisition during prolonged testing or complex movement scenarios. These constraints may limit the wider adoption of OpenCap for high-precision biomechanical analysis. To address these critical gaps, future research should prioritize three key directions. First, the dimensionality of multi-DOF kinematic outputs should be expanded by optimizing keypoint detection algorithms and increasing the number of tracked anatomical keypoints. Second, large-scale datasets encompassing more diverse populations and movement tasks should be constructed to improve the generalizability of the underlying model and the robustness of data acquisition. Third, the technical iteration and translational implementation of low-cost markerless motion capture technologies should be continuously advanced to reduce the dependence of biomechanical analysis on laboratory-based settings and further expand the accessibility and application boundaries of human biomechanical analysis.

Ultimately, the continued development of OpenCap and analogous low-cost markerless motion capture technologies is expected to promote broader access to high-fidelity biomechanical analysis and to provide an important foundation for evidence-based innovation in clinical rehabilitation, sports injury prevention, and human performance optimization.

## Data Availability

The original contributions presented in the study are included in the article/Supplementary Material, further inquiries can be directed to the corresponding authors.
